# Cholangitis and Budd Chiari Syndrome as Complications of Simple Cystic Liver Disease

**DOI:** 10.1155/1993/47873

**Published:** 1993

**Authors:** A. J. Johnstone, L. W. Turnbull, P. L. Allan, O. J. Garden

**Affiliations:** 1 University Department of Surgery Royal Infirmary of Edinburgh 1 Lauriston Place Edinburgh EH3 9YW UK; 2 University Department of Radiology Royal Infirmary of Edinburgh 1 Lauriston Place Edinburgh EH3 9YW UK

## Abstract

We report the case of a 63 year old woman who developed the complications of cholangitis and Budd Chiari syndrome secondary to polycystic disease of the liver. The two complications were not present simultaneously, and both resolved after decompression of the liver cysts.


*HPB Surgery*, 1993, Vol. 6, pp. 223–228
Reprints available directly from the publisher
Photocopying permitted by license only

© 1993 Harwood Academic Publishers GmbH
Printed in the United States of America

### CASE REPORT

### CHOLANGITIS AND BUDD CHIARI SYNDROME AS COMPLICATIONS OF SIMPLE CYSTIC LIVER DISEASE

A.J. JOHNSTONE[1], L.W. TURNBULL[2], P.L. ALLAN[2] and O.J. GARDEN[1]
*University Departments of Surgery[1] and Radiology[2], Royal Infirmary of Edinburgh, 1 Lauriston Place, Edinburgh, EH3 9YW, UK*

*(Received 13 May 1991)*

We report the case of a 63 year old woman who developed the complications of cholangitis and Budd Chiari syndrome secondary to polycystic disease of the liver. The two complications were not present simultaneously, and both resolved after decompression of the liver cysts.

KEY WORDS: Liver cysts, cholangitis, Budd Chiari syndrome

### CASE REPORT

In October 1988, a 63 year old female patient was admitted with fever, rigors and jaundice. Examination revealed hepatomegaly. Investigation demonstrated cholestatic jaundice (Table 1) with normal coagulation. Tests for infective and autoimmune causes of liver disease were negative. Ultrasound scanning and computerised tomography (CT scan) demonstrated multiple liver cysts, a normal gallbladder and intrahepatic duct dilatation. Endoscopic retrograde cholangiopancreatography failed to outline the biliary tree but the pancreatogram was normal.

This acute episode settled on treatment with antibiotics and the patient declined further investigation or treatment. In March 1989, she experienced a further episode of cholangitis associated with abdominal distension. Examination revealed marked hepatosplenomegaly and ascites. Ultrasound and CT scans demonstrated multiple liver cysts, the largest measuring 17 cm × 15 cm × 15 cm and situated centrally, occupying the quadrate lobe (segment IV). The scans also showed compression of the porta hepatis by the largest cyst, intrahepatic duct dilatation and mild splenomegaly.

Laparotomy was performed and 2 litres of ascitic fluid removed. The majority of the liver cysts, including the largest, were de-roofed and marsupialised with the exception of a few small intrahepatic cysts shown on peroperative ultrasonography. Liver function tests steadily improved in the post-operative period (Table 1) and

Address correspondence to: Mr O. J. Garden, Senior Lecturer, University Department of Surgery, Royal Infirmary of Edinburgh, 1 Lauriston Place, Edinburgh, EH3 9YW, UK

the ascites and peripheral oedema resolved with diuretics and salt restriction. A follow up CT scan showed no increase in the size of the remaining liver parenchyma.

The patient was discharged home and remained well until November 1989, at which time she became aware of increasing abdominal distension. Examination revealed marked hepatomegaly and moderate ascites. The full blood count, blood coagulation, liver function tests and serum albumin were normal (Table 1). A further abdominal CT scan showed apparent recurrence of the largest cyst in the quadrate lobe and marked hypertrophy of the caudate lobe (Figures 1 and 2). The inferior vena cava was compressed (Figure 1) and the azygos, hemiazygos and perisplenic veins were dilated. A Doppler ultrasound scan demonstrated blood flow in the right and middle hepatic veins but normal cardiac and respiratory variations were not seen. The large recurrent cyst contained debris and septae originating from the cyst wall. A sample of the cyst contents and ascitic fluid were obtained by aspiration under ultrasound guidance. The cyst fluid contained a few pus cells but no organisms. The protein content of the fluid was 47 g/L and that of the ascitic fluid was 44 g/L. No cells or organisms were identified in the ascitic fluid.

**Table 1**

| | Oct '88 | Mar '89 | Nov '89 |
|---|---|---|---|
| Bilirubin (umol/L) | 40 | 40 | 12 |
| Alkaline phosphatase (U/L) | 528 | 168 | 70 |
| Alanine aminotransferase (U/L) | — | 13 | 10 |
| Gamma glutamyl transferase (U/L) | 804 | 43 | 22 |
| Albumin (g/L) | 38 | 25 | 39 |

The clinical picture was that of a Budd Chiari syndrome caused by inferior vena caval and hepatic vein occlusion secondary to the pressure effects of the reformed cyst in the quadrate lobe.

The patient underwent laparotomy and drainage of 6 litres of ascitic fluid, extended left hepatectomy with preservation of the caudate lobe, and cholecystectomy. Following drainage of the large cyst, peroperative ultrasonography revealed filling of the inferior vena cava and marked distension of the right hepatic vein. Histology revealed mild portal fibrosis and mild cholangitis in keeping with compression by the cysts; the gallbladder was normal. The patient made an uneventful recovery, being discharged on the tenth post-operative day. The patient remains asymptomatic to date, with no evidence of ascites or peripheral oedema.

### DISCUSSION

Congenital liver parenchymal cysts are uncommon; solitary cysts are more common than multiple cysts[1–3] which may be found in isolation or in association with

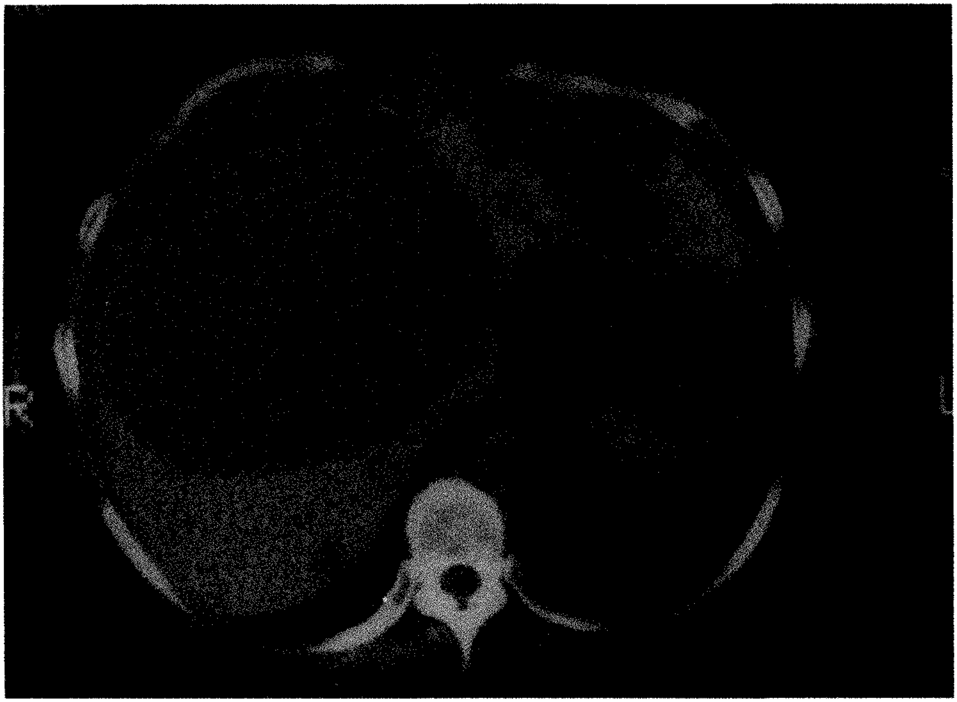

**Figure 1** CT scan showing marked ascites (A), recurrent liver cyst (C) and compression of the inferior vena cava (V).

polycystic disease of the kidney[4–6]. When associated with renal disease, the long-term outcome is frequently determined by renal function[7]. Hepatic cysts are rarely symptomatic but complications such as haemorrhage, rupture, infection and complications of portal hypertension have been reported[3,8–13]. Liver failure is uncommon and in those patients who have been submitted to liver transplantation the cardinal indication has been intractable symptoms. In the symptomatic patient the results of percutaneous aspiration[14] have been disappointing and most success has been reported by surgical manoeuvres, including de-roofing and marsupialisation of the cyst wall.

The present case is noteworthy in that the association between obstructive jaundice and hepatic cysts is rare[1,15,16]. This patient's recurrent cholangitis could not be attributed to gallstones or bile duct pathology and this is the second occasion that this complication has been reported in association with hepatic cysts[17].

The protein content of the ascitic fluid was not determined at the time of the patient's first laparotomy but it seems likely that there was a degree of hepatic vein obstruction at the time of presentation. Her subsequent clinical course, the protein content of the ascitic fluid and the findings on Doppler ultrasonography support the picture of a Budd Chiari syndrome. In retrospect, it might have been preferable to have undertaken hepatic resection at the initial procedure in view of the central position of the cyst and this view is supported by the resected specimen. Hepatic transplantation was not considered in this individual because of the well preserved liver function.

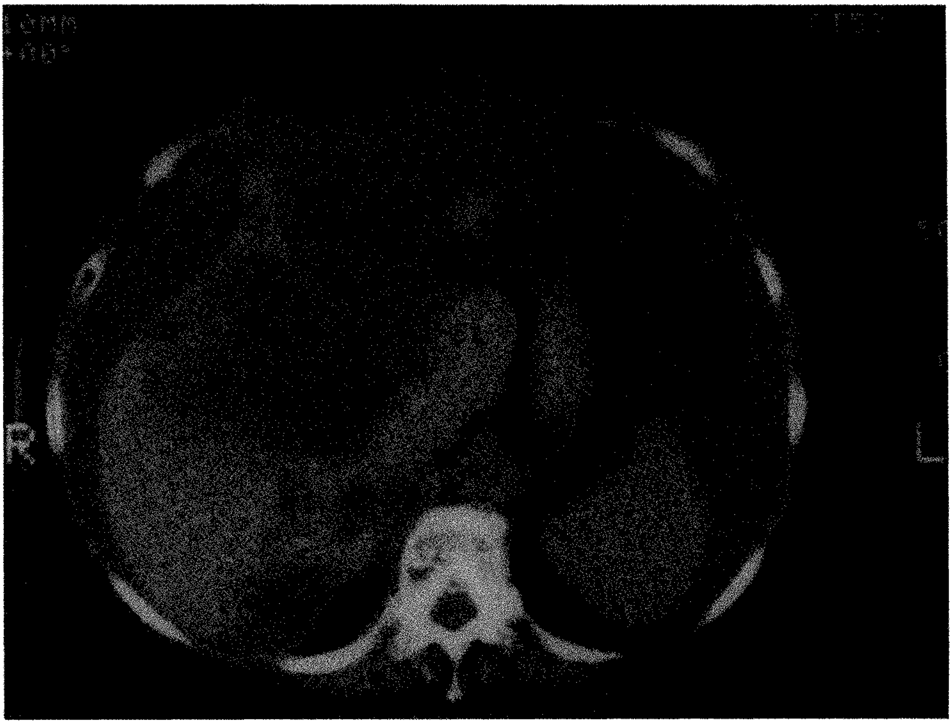

**Figure 2** CT scan showing caudate lobe hypertrophy (CL).

### *References*

1. Sanfelippo, P.M., Beahrs, O.H. and Weiland, L.H. (1974) Cystic disease of the liver. *Annals of Surgery*, **179**, 922–925
2. Rosenburg, G.V. (1956) Solitary non-parasitic cysts of the liver. *American Journal of Surgery*, **91**, 441–444
3. Hadad, A.R., Westbrook, K.C., Graham, G.G., Morris, W.D. and Campbell, G.S. (1977) Symptomatic non-parasitic liver cysts. *American Journal of Surgery*, **134**, 739–744
4. Feldman, M. (1958) Polycystic disease of the liver. *American Journal of Gastroenterology*, **29**, 83–86
5. Kwok, M.K. and Lewin, K.J. (1988) Massive hepatomegaly in adult polycystic liver disease. *American Journal of Surgical Pathology*, **12**, 321–324
6. Thomsen, H.S., Thaysen, J.H. (1988) Frequency of hepatic cysts in adult polycystic kidney disease. *Acta Medica Scandinavia*, **224**, 381–384
7. Melwick, D.J. (1955) Polycystic liver. *Archives of Pathology*, **59**, 162–172
8. Davis, C.R. (1937) Nonparasitic cysts of liver. *American Journal of Surgery*, **35**, 590–594
9. Morgenstern, L. (1959) Rupture of solitary nonparasitic cyst of the liver. *Annals of Surgery*, **150**, 167–171
10. Ackman, F.D. and Rhea, L.J. (1931) Non-parasitic cysts of the liver: their clinical and pathological aspects. *British Journal of Surgery*, **18**, 648–654
11. Campbell, G.S., Bick, H.D., Paulson, E.P., Lober, P.H., Watson, C.J. and Varco, R.L. (1958) Bleeding esophageal varices with polycystic liver disease. *New England Journal of Medicine*, **259**, 904–910

12. Del Guercio, E., Greco, J., Kim, K.E., Chinitz, J. and Swartz, C. (1973) Esophageal varices in adult patients with polycystic kidney and liver disease. *New England Journal of Medicine*, **289**, 678–679
13. Ratcliffe, P.J., Reeders, S. and Theaker, J.M. (1984) Bleeding oesophageal varices and hepatic dysfunction in adult polycystic kidney disease. *British Medical Journal*, **288**, 1330–1331
14. Jones, W.L., Mountain, J.C. and Warren, K.W. (1974) Symptomatic non-parasitic cysts of the liver. *British Journal of Surgery*, **61**, 118–123
15. Howard, R.J., Hanson, R.F. and Delaney, J.P. (1976) Jaundice associated with polycystic liver disease. *Archives of Surgery*, **111**, 816–817
16. Wittig, J.H., Burns, R. and Longmire, W.P. (1978) Jaundice associated with polycystic liver disease. *American Journal of Surgery*, ,bs>136, 383–386
17. Deutsch, E. (1953) Congenital cystic disease of the kidneys and liver complicated by cholangitis; case report. *Gastroenterology*, **23**, 92–96

(*Accepted by S. Bengmark 28 October 1991*)

### INVITED COMMENTARY

This paper is a very interesting case report describing Budd-Chiari syndrome as a rare complication of nonparasitic liver cysts. Nonparasitic liver cysts usually remain small and asymptomatic, however some of them slowly enlarge with time and may produce chronic symptoms as a result of capsular stretching or compression. Presentations such as, acute hemorrhage, cyst rupture, jaundice and cholangitis due to compression of a duct, torsion of a pedunculated cyst, and infection of cyst fluid, is uncommon in this disease. On the other hand, even in asymptomatic liver cysts, adenocarcinoma arising from a single cyst must always be considered, as we have described in a recent paper in *Gastroenterologica Japonica*, (**26**, 80–89, 1991).

In this case, a large liver cyst of the quadrate lobe was recurrent, producing Budd-Chiari syndrome, in spite of deroofing it at the first laparotomy. In general, the recommended surgical treatment for symptomatic large cysts of the liver has been either removal of the cyst or drainage using very wide deroofing and/or fenestration. If the patients have cysts communicating with the biliary tree, operative methods to be considered include haptic resection or biliary drainage into a Roux-en Y loop of jejunum. Prior to operative treatment of liver cysts, endoscopic retrograde cholangiography or intraoperative cholangiography is necessary to establish if there is a communication between the cyst and the biliary tree, even when the cyst fluid is not bile stained. As described, this case was associated with not only Budd-Chiari syndrome but also cholangitis. For the reason mentioned above, intraoperative cholangiography should have been performed in this case.

We have seen 23 cases of nonparasitic liver cyst during the past fourteen and half years in our department, including one case of adenocarcinoma arising from a cyst. One patient had a huge solitary cyst (17 × 18 × 15cm) in the posterior segment of the liver with significant compression of the inferior vena cava. She demonstrated remarkable venous dilatation of abdominal wall and edema of both legs. The postoperative course was favourable after posterior segmentectomy, with complete relief of the symptoms of inferior vena cava compression. Therefore, if these patients have severe clinical symptoms such as jaundice, edema, or pain, hepatectomy is the best treatment and the first choice.

Ryuji Mizumoto
Professor and Chairman
Department of Surgery
Mie University, School of Medicine
2–174 Edobashi
Tsu City, Mie-Ken 514, Japan

### INVITED COMMENTARY

This case report is important because it represents unusual complications of multiple large liver cysts which if improperly treated could have resulted in death of the patient.

The magnitude of the complications in this patient, including jaundice and ascites, which are rare in simple cysts of the liver, were presumably due to pressure of the large cysts on the bile ducts and on segments of the portal venous system leading to Budd Chiari Syndrome, required unusual judgment and skill in their management.

Jaundice in 2 of 14 cases reported by me was due to the presence of a common duct stone in one patient and to a cluster of simple cysts in the main bile ducts in the other. Neither had ascites or segmental portal obstruction.

The authors raise the legitimate question of whether a left trisegmentectomy would have been the appropriate initial operation, which was done subsequently.

Surgeons have the same privilege of second guessing themselves as do others, but since surgeons are dealing with life or death they exercise this choice with greater gravity and enjoy the options less. The reviewer agrees that the operations were performed in proper sequence.

It has been my experience that the larger the cysts, the larger operation is preferable. Fortunately, major resections of the liver can be done in this setting with no or very acceptable mortality. Occasionally a large hepatic cyst can be completely enucleated. If unroofing is chosen, the dome excised must be generous. Marsupialization is frequently mentioned in combination with unroofing of large cysts of the liver, as alluded to in this paper, but is rarely performed. Marsupialization means suturing the edge of the open cyst to the skin, thus forming a permanent pouch.

### *Reference*

Wm. Lloyd Jones, John C. Mountain and Kenneth W. Warren (1974) Symptomatic Cysts of the Liver, *British Journal of Surgery*, **61**, 118–123

Kenneth W. Warren
125 Parker Hill Avenue
Boston, MA 02120, USA

